# Supplementary material for: Exposure Route Influences Disease Severity in the COVID-19 Cynomolgus Macaque Model
Source: Viruses. 2022 May 10;14(5):1013. doi: 10.3390/v14051013 (PMC9145782; doi:10.3390/v14051013)
Supplement: Supplementary file 1 [file viruses-14-01013-s001.zip › viruses-1683202-supplementary.pdf]

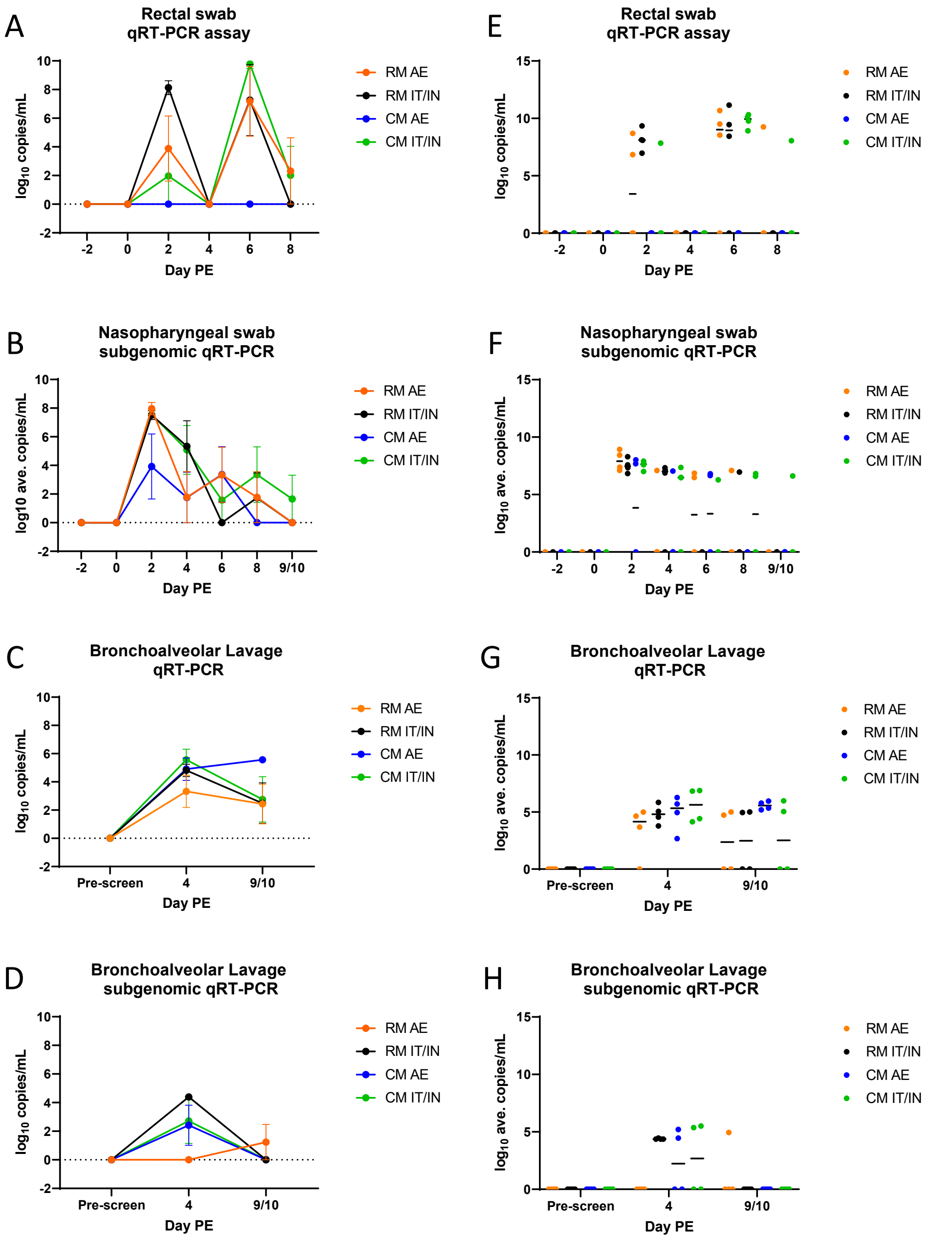

Supplementary Figure S1. Viremia in biosamples from rhesus (RM) and cynomolgus macaques (CM) infected with SARS-CoV-2 by aerosol (AE) or intratracheal/intranasal (IT/IN) exposure by day post-exposure (PE). Viral RNA in rectal swabs (**A, E**) and BAL samples (**C, G**) as detected by RT-PCR. Detection of subgenomic RNA in NP swabs (**B, F**) and BAL samples (**D, H**) using real-time RT-PCR. Data in **A-D** are shown as the mean ± SEM, while individual data points are shown in **E-H** with the black line representing the median for each group.

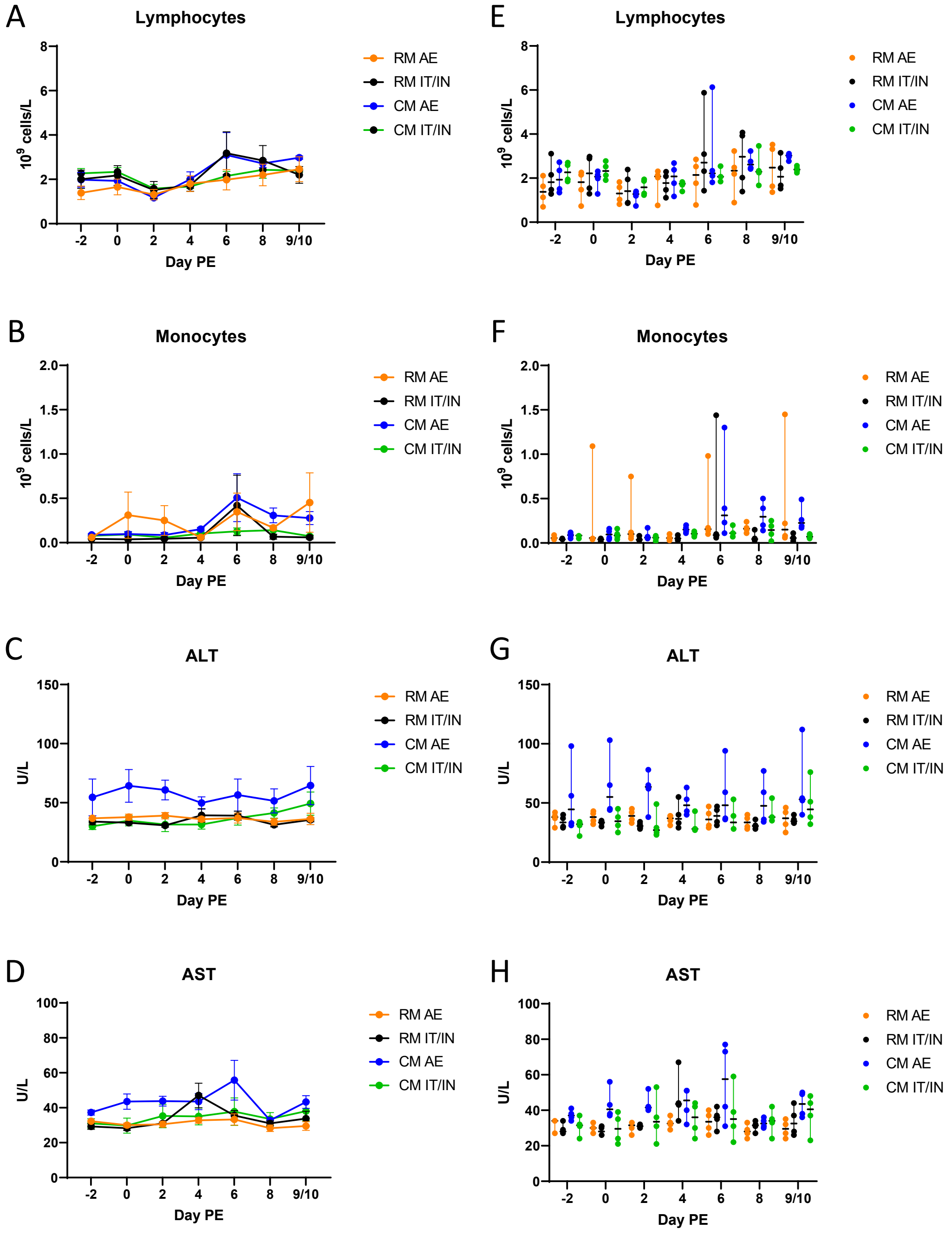

Supplementary Figure S2. Clinical pathology alterations in rhesus (RM) and cynomolgus macaques (CM) infected with SARS-CoV-2 by aerosol (AE) or intratracheal/intranasal (IT/IN) exposure by day post-exposure (PE). Lymphocyte (**A, E**) and monocyte (**B, F**) counts and ALT (**C, G**) and AST (**D, H**) levels over the course of the study

are shown. Data in **A-D** are shown as the mean ± SEM, while individual data points are shown in **E-H** with the black line representing the median for each group.

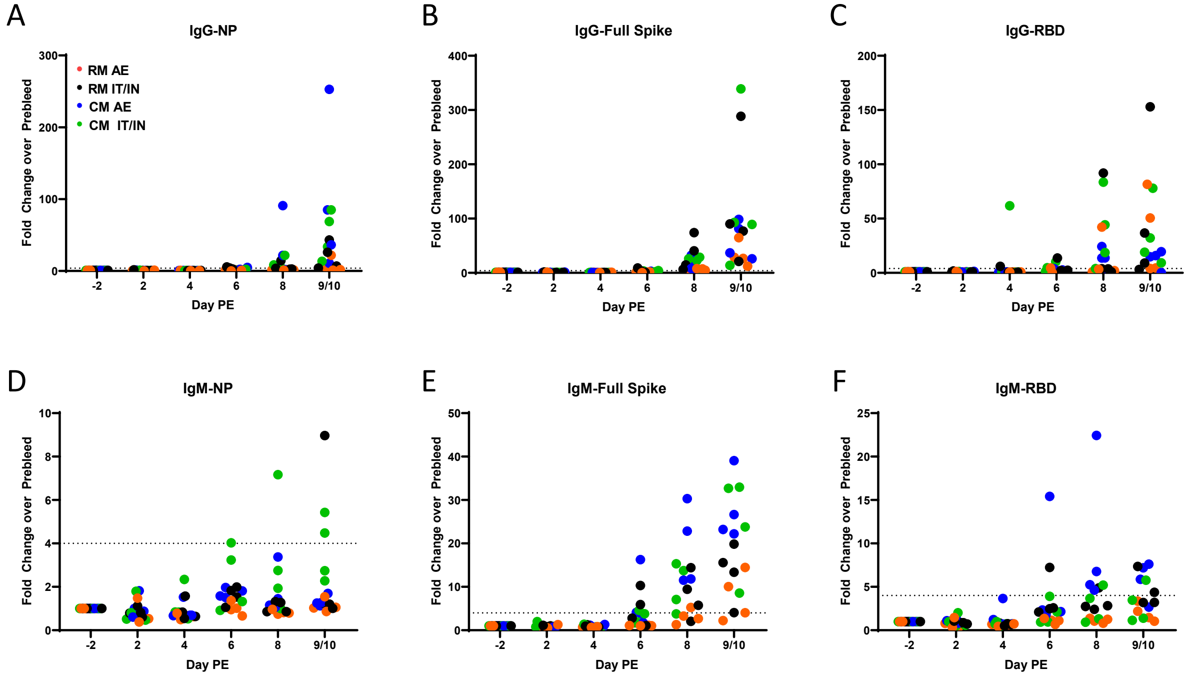

Supplementary Figure S3. Characterization of the IgG and IgM responses in rhesus (RM) and cynomolgus macaques (CM) infected with SARS-CoV-2 by aerosol (AE) or intratracheal/intranasal (IT/IN) exposure by day post-exposure (PE). A Magpix multiplex immunoassay was used to measure the IgG (**A**–**C**) and IgM (**D**–**F**) responses to NP, full spike protein, and RBD of SARS-CoV-2 in serum samples. Results are shown as fold change over pre-bleed (Day -2). The dashed line represents the limit of detection of the assay.

Supplementary Table S1. Summary of histopathology findings in lungs from rhesus (RM) and cynomolgus macaques (CM) infected with SARS-CoV-2 by aerosol (AE) or intratracheal/intranasal (IT/IN) exposure.

		Pulmonary Lesions						
Group	Animal	Inflammation	Type II pneumocyte hyperplasia	Fibrin deposition alveolar lumen (strands)/ septa	Intra-alveolar fibrin/ fibrous aggregated deposition	Septal fibrous change	ISH+	MGNCs[A] present
RM AE	RM AE 1	1	2	0	0	0	No	Yes
	RM AE 2	1	1	0	0	0	No	No
	RM AE 3	1	1	0	0	0	Yes	Rare
	RM AE 4	2	2	0	0	1	Yes	Rare
RM IT/IN	RM IT/IN 1	1	1	0	0	0	Yes	No
	RM IT/IN 2	1	1	0	0	1	No	Rare
	RM IT/IN 3	1	0	0	0	1	No	No
	RM IT/IN 4	1	0	0	0	0	No	No
CM AE	CM AE 1	3	3	3	3	2	Yes	Yes
	CM AE 2	3	3	2	0	2	Yes	Yes
	CM AE 3	3	3	2	0	2	Yes	Yes
	CM AE 4	3	3	3	3	3	Yes	Yes
CM IT/IN	CM IT/IN 1	2	2	1	0	2	Yes	Yes
	CM IT/IN 2	2	2	2	0	2	Yes	Yes
	CM IT/IN 3	3	3	2	1	2	Yes	Yes
	CM IT/IN 4	2	2	1	1	1	No	Yes

[A] MGNCs = multinucleated giant cells

Scoring Key	
0	None
1	Minimal
2	Mild
3	Moderate
4	Marked
5	Severe

Supplementary Table S2. Summary of histopathology findings in nasal turbinates from rhesus (RM) and cynomolgus macaques (CM) infected with SARS-CoV-2 by aerosol (AE) or intratracheal/intranasal (IT/IN) exposure.

		Inflammation			
**Group**	**Animal**	**Lymphoplasmacytic**	**Neutrophilic**	**Ulceration**	**Edema**
RM AE	RM AE 1	3	3	No	2
	RM AE 2	2	2	No	2
	RM AE 3	2	2	No	0
	RM AE 4	2	2	No	2
RM IT/IN	RM IT/IN 1	2	2	No	1
	RM IT/IN 2	3	3	No	2
	RM IT/IN 3	2	2	No	1
	RM IT/IN 4	3	3	No	2
CM AE	CM AE 1	2	2	No	0
	CM AE 2	2	2	No	2
	CM AE 3	2	2	Yes	2
	CM AE 4	3	3	No	2
CM IT/IN	CM IT/IN 1	2	2	No	0
	CM IT/IN 2	2	2	No	2
	CM IT/IN 3	2	2	No	1
	CM IT/IN 4	2	2	No	2

Scoring Key	
0	None
1	Minimal
2	Mild
3	Moderate
4	Marked
5	Severe

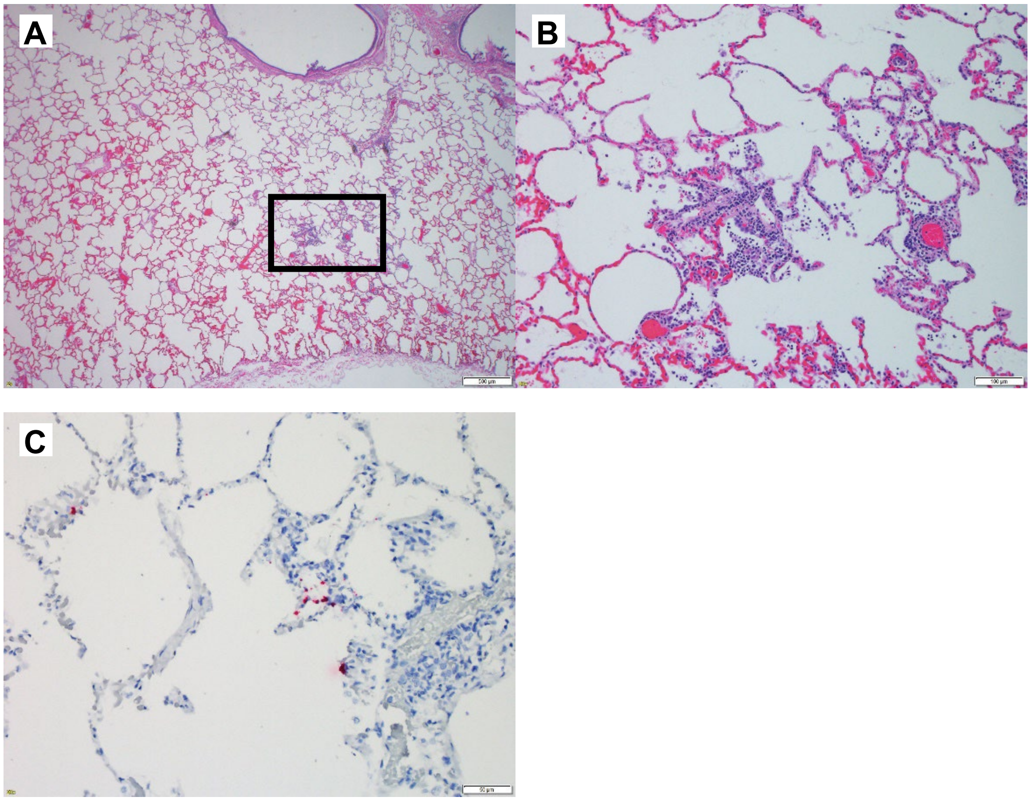

Supplementary Figure S4. Representative pathology in the rhesus macaque (RM) aerosol exposure (AE) group. Images are shown from RM AE 3. (**A**) Lung, left cranial lobe, central: Multifocal minimal interstitial pneumonia, 2x, H&E. (**B**) Lung, higher magnification of boxed area in A: Multifocal minimal inflammation surrounding vessels, expanding alveolar septa and extending into alveolar lumen with minimal type II pneumocyte hyperplasia, 10x, H&E. (**C**) Lung: ISH positive in areas of inflammation, 20x, RNA probe for SARS-CoV-2.

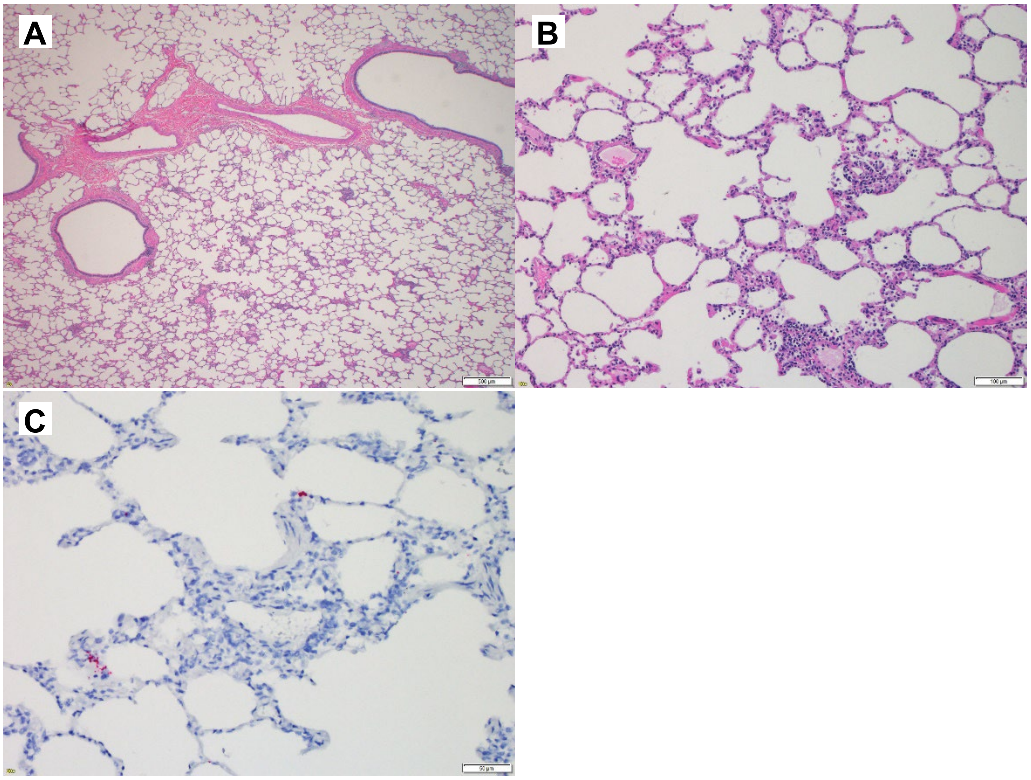

Supplementary Figure S5. Representative pathology in the rhesus macaques (RM) intratracheal/intranasal (IT/IN) exposure group. Images shown are from RM IT/IN 1. (**A**) Lung, right caudal lobe, peripheral: Multifocal minimal interstitial pneumonia, 2x, H&E. (**B**) Lung, higher magnification of boxed area in A: Multifocal minimal inflammation surrounding vessels, expanding alveolar septa and extending into alveolar lumen with minimal type II pneumocyte hyperplasia, 10x, H&E. (**C**) Lung: ISH positive in areas of inflammation, 20x, RNA probe for SARS-CoV-2.

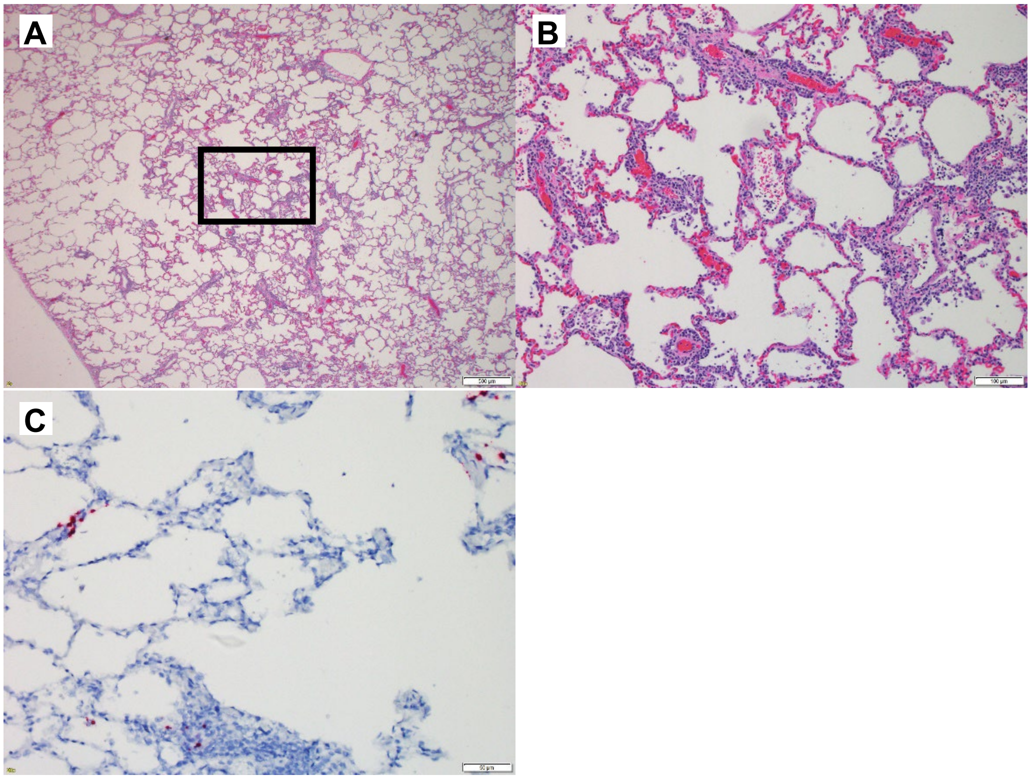

Supplementary Figure S6. Representative pathology in the cynomolgus macaques (CM) intratracheal/intranasal (IT/IN) exposure group. Images shown are from CM IT/IN 1. (**A**) Lung, right caudal lobe, central: Multifocal mild interstitial pneumonia, 2x, H&E. (**B**) Lung, higher magnification of boxed area in A: Multifocal mild inflammation surrounding vessels, expanding alveolar septa and extending into alveolar lumen with mild type II pneumocyte hyperplasia, 10x, H&E. (**C**) Lung: ISH positive in areas of inflammation, 20x, RNA probe for SARS-CoV-2.

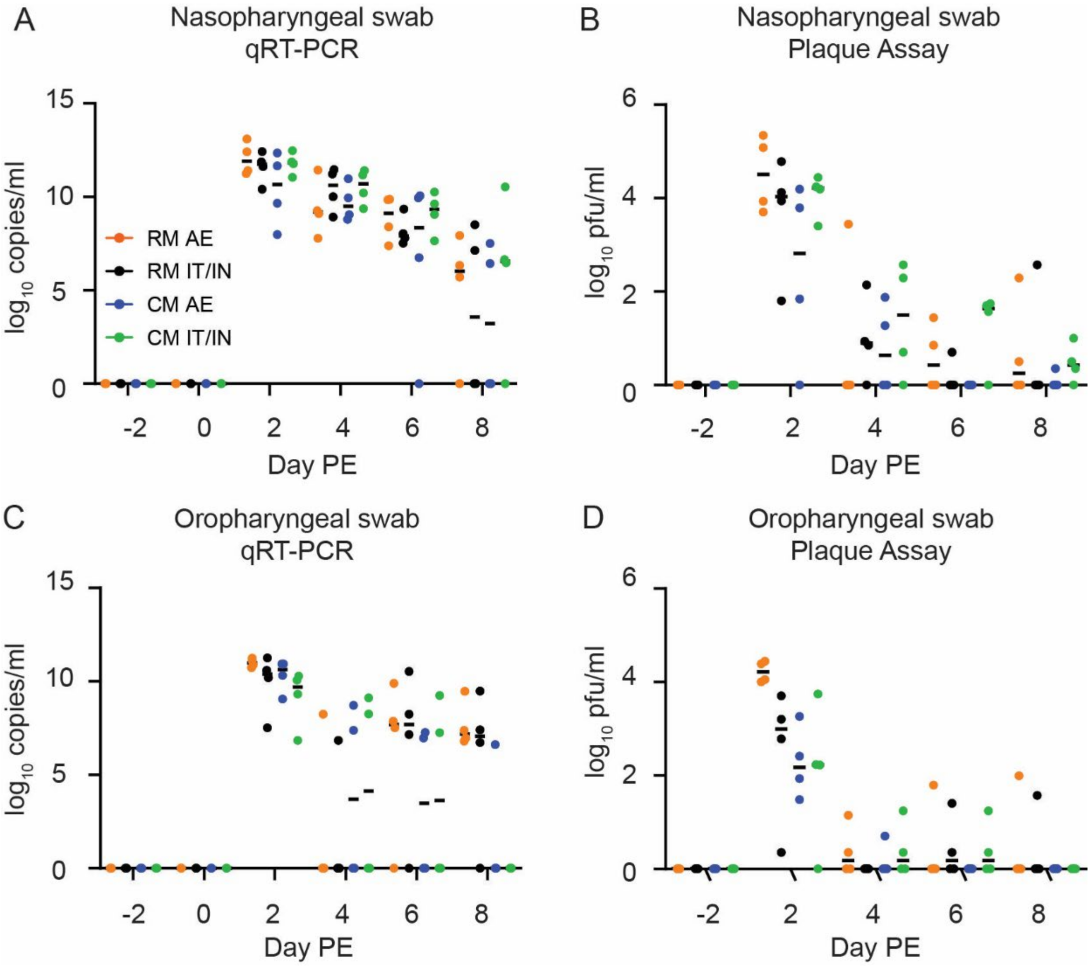

Supplementary Figure S7. Infection of rhesus (RM) and cynomolgus macaques (CM) with SARS-CoV-2 by aerosol (AE) or intratracheal/intranasal (IT/IN) exposure. Detection of viral RNA in NP swabs (**A**) and OP swabs (**C**) by qRT-PCR. Detection of infectious virus by plaque assay in NP swabs (**B**) and OP swabs (**D**). Individual data points are shown with the black line representing the median for each group.

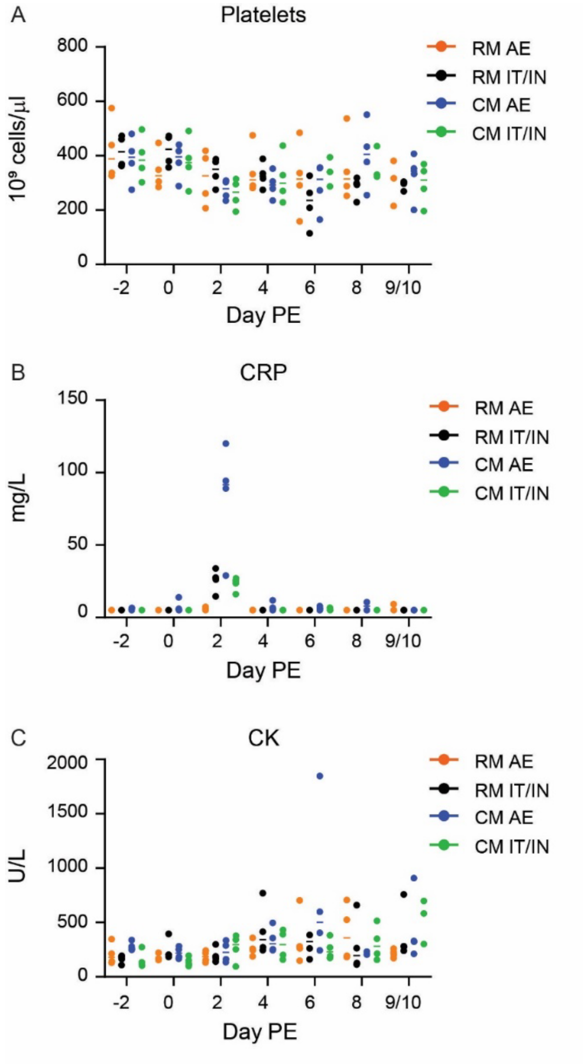

Supplementary Figure S8. Clinical pathology in rhesus (RM) and cynomolgus macaques (CM) infected with SARS-CoV-2 by aerosol (AE) or intratracheal/intranasal (IT/IN) exposure. Levels of platelets (**A**), CRP (**B**), and CK (**C**) over the course of the study. Individual data points are shown with the black line representing the median for each group.

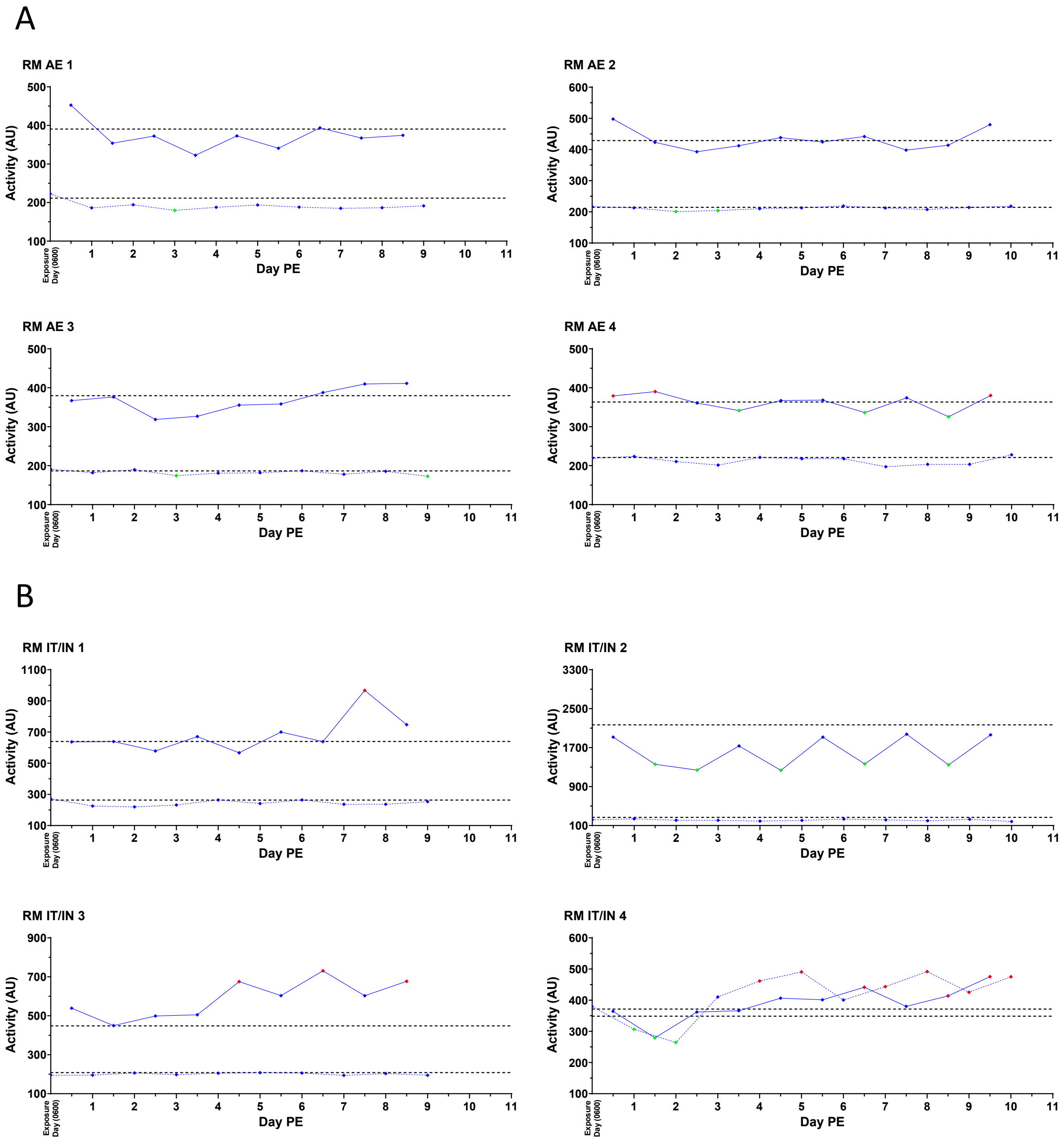

Supplementary Figure S9. Changes in activity levels of rhesus macaques (RM) infected with SARS-CoV-2 by aerosol (AE) (**A**) or intratracheal/intranasal (IT/IN) (**B**) exposure. The 12-hour average activity levels were calculated for each animal. Daytime (0600-1800) values are shown as solid blue lines while nighttime (1800-0600) values are shown as dotted blue lines. The baseline average values for daytime (upper line) and nighttime (lower line) are shown as black dotted lines. Statistically significant increases in activity (+3 SD) are denoted as red diamonds, while significant decreases (-3 SD) appear as green diamonds. Blue diamonds (<3 SD) are not significant.

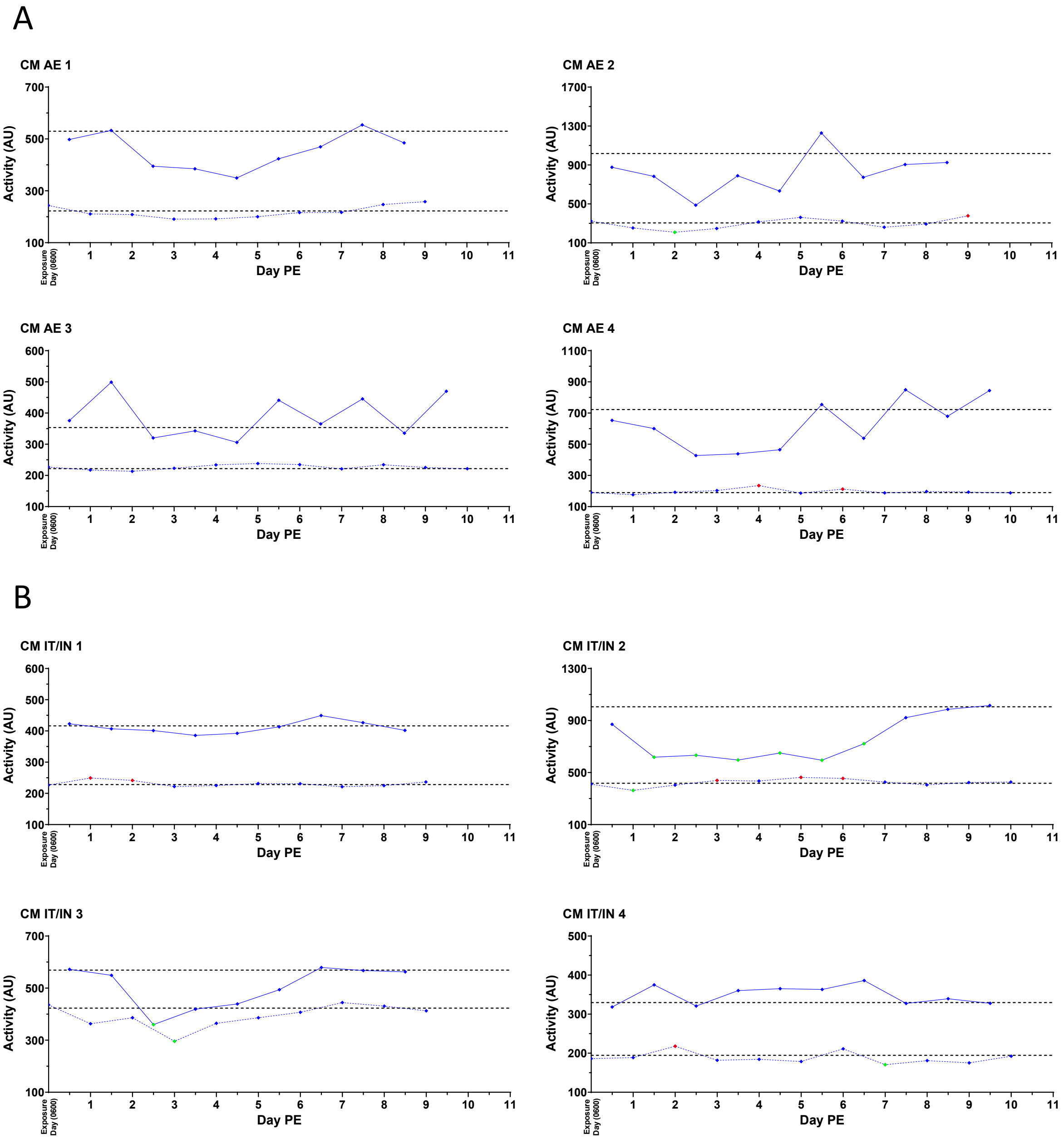

Supplementary Figure S10. Changes in activity levels of cynomolgus macaques (CM) infected with SARS-CoV-2 by aerosol (AE) (**A**) or intratracheal/intranasal (IT/IN) (**B**) exposure. The 12-hour average activity levels were calculated for each animal. Daytime (0600-1800) values are shown as solid blue lines while nighttime (1800-0600) values are shown as dotted blue lines. The baseline average values for daytime (upper line) and nighttime (lower line) are shown as black dotted lines. Statistically significant increases in activity (+3 SD) are denoted as red diamonds, while significant decreases (-3 SD) appear as green diamonds. Blue diamonds (<3 SD) are not significant.
